# Supplementary material for: HIVprotI: an integrated web based platform for prediction and design of HIV proteins inhibitors
Source: J Cheminform. 2018 Mar 9;10:12. doi: 10.1186/s13321-018-0266-y (PMC5845081; doi:10.1186/s13321-018-0266-y)
Supplement: Supplementary file 2 — Additional file 2. Source code of HIVProtI web server. [file 13321_2018_266_MOESM2_ESM.zip › HIVprotI_Source-code/hivprotI/weka-3-6-6/doc/weka/gui/sql/package-frame.html]

weka.gui.sql


weka.gui.sql

Classes 
  
ConnectionPanel
  
DbUtils
  
InfoPanel
  
InfoPanelCellRenderer
  
Messages
  
QueryPanel
  
ResultPanel
  
ResultSetHelper
  
ResultSetTable
  
ResultSetTableCellRenderer
  
ResultSetTableModel
  
SqlViewer
  
SqlViewerDialog
